# Supplementary material for: High-Throughput Separation of Long DNA in Deterministic Lateral Displacement Arrays
Source: Micromachines (Basel). 2022 Oct 17;13(10):1754. doi: 10.3390/mi13101754 (PMC9611613; doi:10.3390/mi13101754)
Supplement: Supplementary file 1 [file micromachines-13-01754-s001.zip › micromachines-1940718-supplementary.pdf]

Electronic Supplementary Materials

High-throughput Separation of Long DNA in Deterministic Lateral Displacement Arrays

Oskar E. Ström[1,2], Jason P. Beech[1,2], and Jonas. O. Tegenfeldt[1,2*]

[1]NanoLund, Lund University, Lund, Sweden

[2]Division of Solid State Physics and NanoLund, Physics Department, Lund University, PO Box 118, 221 00, Lund, Sweden

*Corresponding author. Telephone: +46 46 222 8063. E-mail: jonas.tegenfeldt@ftf.lth.se

1. DLD Array Designs

Table S1. DLD array parameters are given for the two devices that were used.

Device #	Measured gap size, G (µm)	Measured pillar diameter, D_{post} (µm)	Channel Depth, h (µm)	Array Length (mm)	Array width (mm)	N	θ (°)	Calculated Critical Diameter, D_c (µm)
1	2.8	14.7	13	22.9	540	50	1.2	0.60
2	2.24	9.5	9.6	3.6	205	20	2.9	0.74

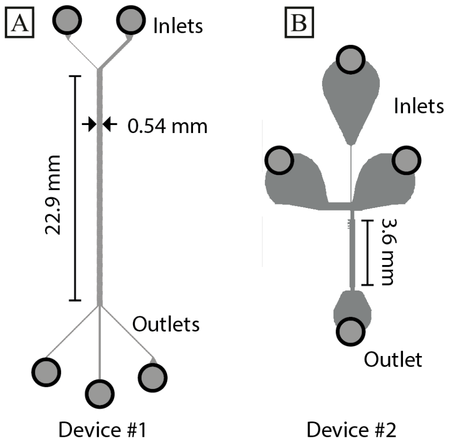

Figure S1. Top views of the DLD device designs #1 (A) and #2 (B). The black circles mark the punched access holes for the inlets and outlets.

2. DNA samples used in our work

For the staining of the DNA in dual-color experiments, we observe cross-migration of the dyes between the samples approximately 30 minutes after mixing. Thus, we made sure that all the imaging was performed within a 30-minute time frame.

Table S2. Overview of DNA samples used. TE stands for Tris EDTA, BME for β- mercaptoethanol, $\boldsymbol{I}$ is the ionic strength (calculated according to Iarko *et al.* [1] with a relative error below 0.6%), $\boldsymbol{w_{eff}}$ is the polymer effective width, $\boldsymbol{l_p}$ is the persistence length, $\boldsymbol{L}$ is the contour length, $\boldsymbol{R_g}$ is the radius of gyration and $\boldsymbol{C^*}$ is the overlap concentration which is defined as the concentration corresponding to one DNA molecule within a volume of a sphere with radius $\boldsymbol{R_g}$. $\boldsymbol{\tau_z}$ is the Zimm relaxation time [2]. $\boldsymbol{R_g}$ is estimated using $\boldsymbol{R_g} \approx \boldsymbol{R_F}/\sqrt{\boldsymbol{6}}$ where $\boldsymbol{R_F}$ is the Flory radius. The calculations of $\boldsymbol{l_p}$, $\boldsymbol{w_{eff}}$ and $\boldsymbol{R_F}$ are described by Reisner *et al.* [3]. The calculation of $\boldsymbol{C^*}$ is described by Doi [4]. The rows colored in green highlight the samples with a relatively low salt concentration. For all entries, T = 21.8°C and a dye to base pair ratio of 1:10 has been assumed. The corresponding figures the samples are presented in is listed in the column "'Fig.".

Fig.	Sample	Size (kbp)	Buffer	I (mM)	l_p (nm)	w_{eff} (nm)	L (µm)	R_g (µm)	C^* (µg/mL)	τ_{Zimm} (s)
3A	5 kbp	5	2.2x TE, 3% BME	21	51.5	7.37	2.0	0.15	390	0.048
3A, 3D	5 kbp	5	4.7x TE, 3% BME	41	50.8	4.80	2.0	0.14	510	0.047
3A, 3D	5 kbp	5	5.0x TE, 3% BME	44	50.7	4.63	2.0	0.13	530	0.047
2(A-C)	DNA ladder lower limit	0.25	1.0x TE	6.1	55.3	16.2	0.1	0.030	2300	-
2B	DNA ladder lower limit	0.25	1.2x TE	7.3	54.4	14.5	0.1	0.030	2500	-
2A	DNA ladder lower limit	0.25	1.8x TE	11	52.9	11.2	0.1	0.030	3000	-
2C	DNA ladder lower limit	10	2.0x TE	12	52.6	10.4	0.1	0.030	3100	-
2(A-C)	DNA ladder upper limit	10	1.0x TE	6.1	55.3	16.2	3.9	0.26	140	0.18
2B	DNA ladder upper limit	10	1.2x TE	7.3	54.4	14.5	3.9	0.26	150	0.18
2a	DNA ladder upper limit	10	1.8x TE	11	52.9	11.2	3.9	0.24	180	0.17
2C	DNA ladder upper limit	10	2.0x TE	12	52.6	10.4	3.9	0.24	190	0.17
3B	λ-DNA	48.5	3.9x TE, 3% BME	35	50.9	5.34	19	0.53	85	2.6

3B	λ-DNA	48.5	4.9x TE, 3% BME	41	50.8	4.86	19	0.52	90	2.6
3B	λ-DNA	48.5	5x TE, 3% BME	44	50.7	4.63	19	0.51	93	2.6
1	λ-DNA	48.5	0.93x TE	5.2	56.2	17.9	19	0.69	39	3.0
1, 2(A-C)	λ-DNA	48.5	1.0x TE	6.1	55.3	16.2	19	0.67	42	2.9
2B	λ-DNA	48.5	1.2x TE	7.3	54.4	14.5	19	0.65	45	2.8
2A	λ-DNA	48.5	1.8x TE	11	52.9	11.2	19	0.62	54	2.7
2C	λ-DNA	48.5	2.0x TE	12	52.6	10.4	19	0.61	57	2.7
3C, 3D, 5A	T4-DNA	166	4.9x TE, 3% BME	43	50.8	4.68	65	1.1	36	23
3C, 3D, 5A, 6, 7	T4-DNA	166	5.0x TE, 3% BME	44	50.7	4.63	65	1.1	36	23
3C, 5(A-C)	T4-DNA	166	0.26x TE	1.6	70.3	36.7	65	1.7	8.6	34
S3	T4-DNA	166	0.68x TE	4.1	57.9	20.7	65	1.5	14	27
1	T4-DNA	166	0.85	5.2	56.2	17.9	65	1.4	15	26
4	T4-DNA	166	0.93x TE	5.7	55.7	16.9	65	1.4	16	25
1, S3	T4-DNA	166	1,0x TE	6.1	55.3	16.2	65	1.4	16	25

3. Image Processing

Python was used to process the fluorescence videography data. The outlet distributions were obtained by integrating the fluorescence intensity across five lines between the pillar rows at the end of the DLD array. The typical integration time was 60 s per pressure value. Note that for the higher pressures (2 bar and 3 bar), the integration times were shorter to reduce sample loss. The intensity signal was made discrete by summing up the total intensity between every two pillars along the line. As a final step, the data of the same sample was normalized so that the total area was the same in each plot.

Images were corrected for two types of errors. Firstly, the illumination had a heterogeneous spatial intensity over the field of view. This signal was measured and its inverse was multiplied with the fluorescent micrograph data. Secondly, the overall background signal was subtracted by locally comparing to the intensity of an area outside the channel.

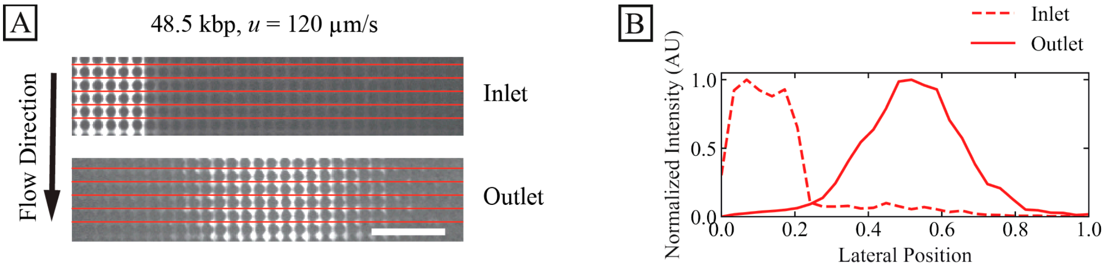

Figure S2. Typical inlet and outlet distributions. Panel A shows fluorescence micrograph snapshots of the array inlet and outlet regions. Panel B shows the corresponding inlet and outlet distributions. These are extracted by spatial and temporal averaging of the intensity along rows of pixels situated between rows of pillars. Each distribution is based on an average of five consecutive rows of pillars as indicated by the red lines in panel A. The data for this example is based on the following sample: 48.5 kbp DNA, u = 120 μm/s, C = 6 μg/mL and a buffer of 4.6x TE and 3% BME. Scale bar is 100 μm.

4. Re-run of processed DNA

To characterize any fragmentation of the DNA strands in the device, DNA from the outlet was re-inserted at the inlet of the same array after being processed. A solution of 12 μg/mL T4-DNA in a buffer of 1x Tris EDTA was run across an array at the highest applied pressure (3 bar) for 15 minutes, see Figure S3. The measured volumetric flow rate through the sample inlet corresponds to approximately Q = 20±2 μL/h, u = 29±3 mm/s and a throughput of 200±20 ng/h. The resulting DNA from two of the outlets (outlets 1 and 3 in Figure S3) were collected and then sequentially run again in the same device.

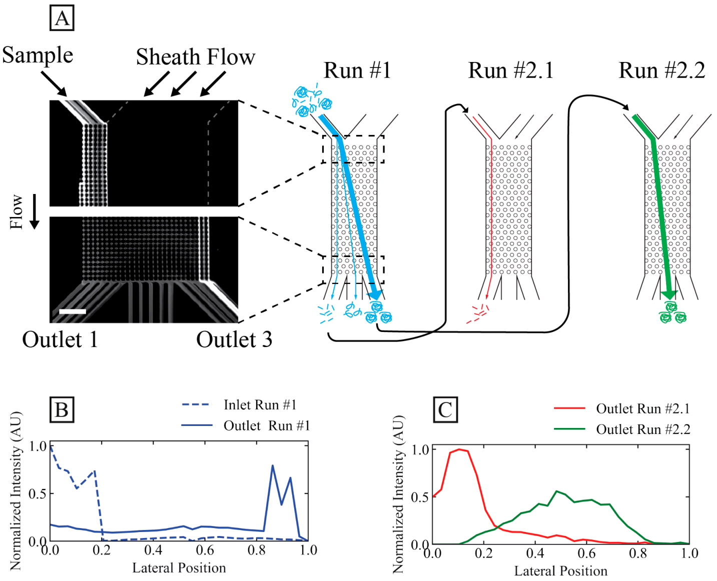

Figure S3. Re-run of a 166 kbp long sample (T4-DNA) at applied pressure differences of 3 bar. The sample, 12 μg/mL (C/C^* ≈ 0.88) T4-DNA in 1x TE buffer, was first processed with the major fraction of the sample being fully displaced and ending up in outlet 3. This corresponds to run #1, see panel (A) for fluorescence micrograph time averages and (B) for the lateral distribution at the inlet and the outlet of the array. Note that some shorter fragments ended up in outlet 1. The samples from outlet 1 and outlet 3 were collected and were re-inserted into the inlet, one sample at a time. The sample from outlet 3 (run #2.1) resulted in a significantly displaced sample. The sample from outlet 1 (run #2.2) was exhibiting no displacement. The scale bar is 100 μm.

If the processed DNA would be significantly fragmented, the displaced fraction would not displace again when re-run. While the level of displacement is not exactly the same as in the first run, this is most likely due to the sample being diluted (approximately 2×) when exiting to the outlet. As shown in Figure S3C, dilute 166 kbp does only displace to a lateral outlet position of approximately 0.3 at the maximum applied pressure (3 bar, corresponding to 29 mm/s). In Figure S3, the displacement reaches a lateral outlet position of 0.5. If the sample would have been significantly fragmented, the sample would show a lateral distribution similar to that of the dilute 5 kbp or 48.5 kbp samples without any displacement in Figure S3A and S3B.

5. Calculation of Purity and Recovery

Purity and recovery are important performance indicators for sorting devices. They can be adjusted and optimized for each particular application by positioning the cutoff for the fractionation at the end of the device. With one cutoff, we can thus expect to divide the mixed sample into two fractions corresponding to small (less deflected) and large (more deflected) molecules. By adjusting where the boundary for collecting the samples is located at the array outlet, we can optimize for either purity or recovery.

We define the purity as the fraction of the desired DNA species exiting into one fraction relative to all DNA exiting that reservoir. We define recovery as the fraction of one DNA species exiting into one fraction relative to all DNA of that species in the two fractions. To calculate the relevant purities and recoveries, we start by considering the normalized intensity curves, see Figure 1 C in the main text. These are probability density functions, ρ, corresponding to the expected lateral position, x, of a specific DNA species at the end of the channel. The fraction of all the considered DNA of that particular species that exits within the interval, (x, $x+dx$) of relative positions is then given by $\rho \times dx$. Together with the concentrations, C, of the different DNA species, we can now calculate the purity and the recovery as a function of DNA species and position of the cutoff. The subscripts λ and T4 refer to the small and large DNA species. The position of the cutoff is denoted by $0 < x_{th} < 1$. We calculate the purities and the recoveries for the small species collected into the small-species fraction, *i.e.* for relative exit positions $0 < x < x_{th}$, and the purities and the recoveries for the large species collected into the large-species fraction, *i.e.* for relative exit positions $x_{th} < x < 1$.

$$Purity\ (\lambda, x_{th}) = \frac{C_\lambda \int_0^{x=x_{th}} \rho_\lambda(x)dx}{C_\lambda \int_0^{x=x_{th}} \rho_\lambda(x)dx + C_{T4} \int_0^{x=x_{th}} \rho_{T4}(x)dx}$$

$$Purity\ (T4, x_{th}) = \frac{C_{T4} \int_{x=x_{th}}^{1} \rho_{T4}(x)dx}{C_\lambda \int_{x=x_{th}}^{1} \rho_\lambda(x)dx + C_{T4} \int_{x=x_{th}}^{1} \rho_{T4}(x)dx}$$

$$Recovery\ (\lambda, x_{th}) = \frac{C_\lambda \int_0^{x=x_{th}} \rho_\lambda(x)dx}{C_\lambda \int_0^{1} \rho_\lambda(x)dx} = \int_0^{x=x_{th}} \rho_\lambda(x)dx$$

$$Recovery\ (T4, x_{th}) = \frac{C_{T4} \int_{x=x_{th}}^{1} \rho_{T4}(x)dx}{C_{T4} \int_0^{1} \rho_{T4}(x)dx} = \int_{x=x_{th}}^{1} \rho_{T4}(x)dx$$

The calculated results for purity and recovery as a function of cut-off based on the data in Figure 1C in the main text are given in Figures S4 and S5.

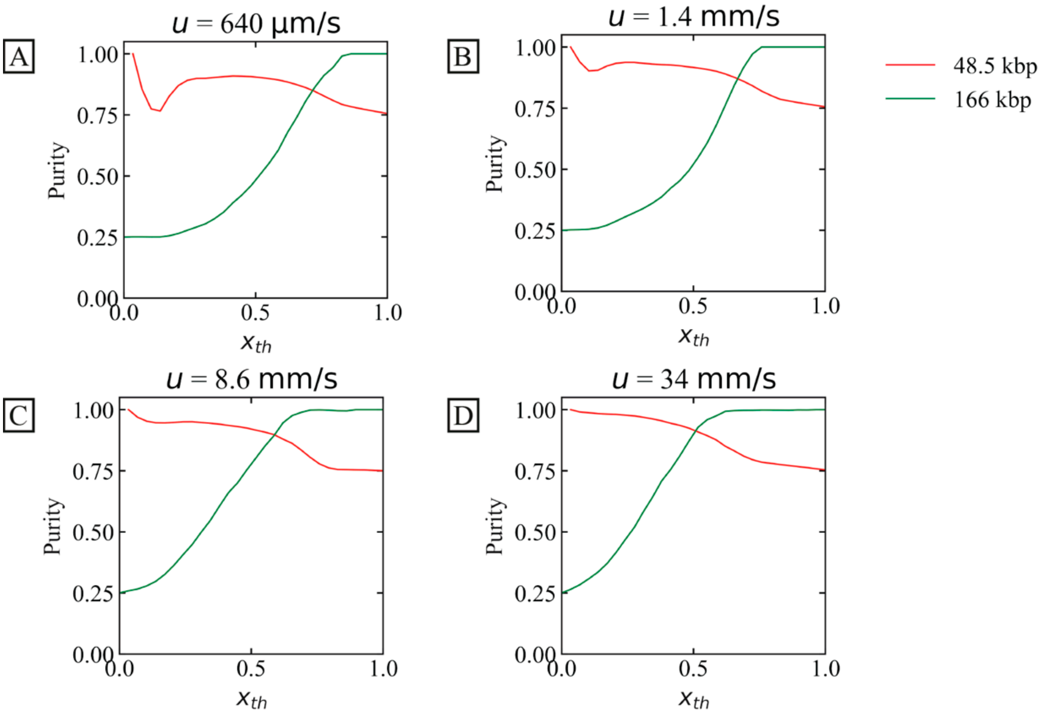

Figure S4. Estimated purities as a function of relative lateral position of cut-off, x_{th}, based on the results of sorting T4-DNA from λ-DNA, see Figure 1 in main text. The cutoff is given as the relative lateral position across the whole width of the device where the sample is divided into two fractions.

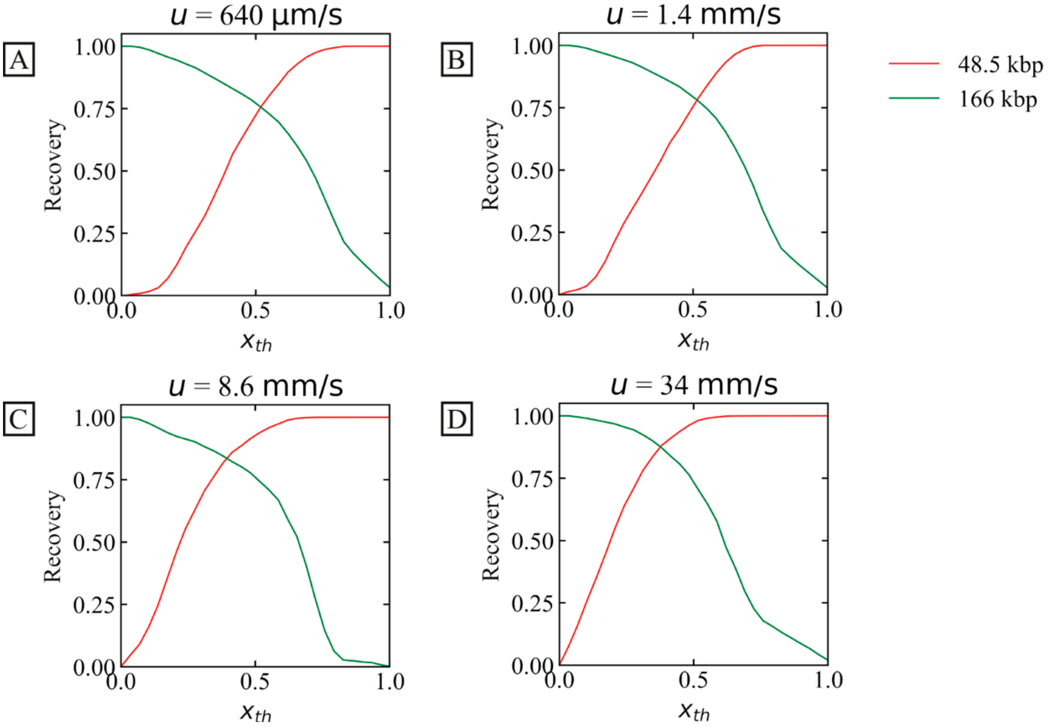

Figure S5. Estimated recoveries as a function of relative lateral position of cut-off, x_{th}, based on the results of sorting T4-DNA from λ-DNA, see Figure 1 in main text. The cutoff is given as the relative lateral position across the whole width of the device where the sample is divided into two fractions.

6. Overview of performance of different sorting devices

In the following we present an overview of the performance that has been demonstrated for different sorting technologies. Note that the sample throughput refers to the total sample that is processed, *i.e.* the input sample.

Table S3. Reported performance indicators for displacing (with DLD) or sorting long DNA (≥ 48.5 kbp) in microfluidic pillar arrays. The table compares our work using optimized conditions to previous work. Note that the techniques are also compared with the conventional gel-based technique pulsed-field gel electrophoresis (PFGE). When the transport mode is electrophoresis, the presented flow rate is an effective flow rate calculated by multiplying the migration velocity with the cross-sectional area of the array (depth × pillar gap × number of gaps).

Reference	Transport Mode	Experiment and sample	Pillar gap size (µm)	Highest Flow or migration velocity (µm/s)	Highest flow rate (sample inlet) (µL/h)	Highest sample through put (ng/h)	Sample Conc. used for highest throughput (µg/mL)
Current work (estimated)	Pressure-driven	166 kbp, 48.5 kbp	2.9	34 000*	24	760	8+24=32
Huang *et al.*, 2004 [5]	Electrophoresis	DLD Electrophoretic separation of bacterial artificial chromosomes (61 kbp & 158 kbp)	3	20	0.04‡	~0.12‡	3
Chen *et al.*, 2015 [6]	Electrophoresis	DLD T4 (166 kbp) displacement & concentrating of DNA	1.7	40 (w\ PEG) ¶	~0.21§	~0.002§	0.01
Wunsch *et al.*, 2019 [7]	Pressure-driven	DLD displacement of 48.5 kbp	0.078 to 0.75	1500#	~0.03	0.03 **	1 to 5
Wunsch *et al.*, 2019. 1024 parallelized arrays [7] †	Pressure-driven	-	0.225	-	900	-	-
Huang *et al.* 2002 [8]	Electrophoresis	DNA prism Electrophoretic separation of 61 kbp to 209 kbp	2	~400 ‡‡	~1	~100	~10
Doggett *et al.* 1992 [9]	Electrophoresis	Pulsed-field gel electrophoresis (PFGE) ††	-	-	0.05 to 0.2	0.4 to 16	80

* Mean flow velocity between the pillars. ‡ Effective value based on an estimate assuming a 0.3 mm wide inlet channel and 5 µm deep device. §Value based on the peak flow velocity 40 µm/s and interpolation based on the corresponding reported flow rates. ¶ Calculated peak velocity between the pillars assuming a parabolic flow profile. The device also only worked with the addition of PEG as a condensation agent. # The reported velocity is based on particle tracking of the DNA molecules. † Wunsch *et al.* connected 1024 of their arrays in parallel to achieve higher throughput. ** Value based on an estimate assuming a 0.5 µm deep device. ‡‡ Value

based on assuming a migration distance of 6 mm. †† Assuming a gel plug of 800 µL (standard Bio-Rad volume), a highest concentration of 80 µg/mL (see [9]) and run times between 4h and a week.

7. Overview of sorting devices based on DLD used for long DNA

In Table S4 we present an overview of the design parameters of DLD devices from a selection of relevant publications.

Table S4. Reported DLD parameters comparing our devices with devices from previous work that have been used to displace long DNA (>20 kbp). The values for *N* are calculated based on the reported displacement angle if not otherwise detailed. Note that device #2 for our work did not work for high flow velocities.

Reference	Experiment and sample	Pillar gap size (µm)	Pillar diameter (µm)	Array type	N	Displacement angle, θ (°)
Current work (Device #1)	Separation of 166 kbp & 48.5 kbp, and more…	2.9	14.7	Parallelogram	50	1.2
Current work (Device #2)	Displacement of 166 kbp	2.3	9.5	Parallelogram	20	2.9
Huang *et al.*, 2004 [5]	Separation of bacterial artificial chromosomes (61 kbp & 158 kbp)	3	5	Rotated square	10	5.7
Chen *et al.*, 2015 [6]	T4-DNA (166 kbp) concentration	1.7	6.3	Rotated square	15	3.8
Wunsch *et al.*, 2019 [7]	Displacement of 48.5 kbp	0.078 - 0.75	~0.15	Parallelogram	10	5.7

8. Supplementary videos

Video S1. Low magnification (10x) fluorescent videographs of high-throughput separation of 48.5 kbp and 166 kbp at an applied pressure difference of 3 bar, corresponding to a mean flow velocity of 32 mm/s in device #1. The two movies were captured in sequence with different filter cubes appropriate to the fluorophores. The frame rate was 9.83 fps and the exposure time was 100 ms. The video corresponds to the data shown in Figure 1 in the main text.

Video S2. Low magnification (10x) fluorescent videographs of separation of long (48.5 kbp) from short DNA (0.25 kbp to 10 kbp) of varying flow velocities in device #1. The two samples were stained in different dyes and their intensities were captured simultaneously using dual-color microscopy (with an Optosplit as described in the main text). The frame rate was 19.3 fps and the exposure times was 50 ms. Note that the bright ring in the lower left corner in the second half of the video is a reflective artefact and does not correspond to DNA. The video corresponds to the data shown in Figure 2 in the main text.

Video S3. Low magnification (2x) fluorescent videograph of an undulating sample trajectory of long DNA (166 kbp) at 23 µg/mL (0.26x TE buffer) in device #1. The field of view is moved across the array. The undulations change character from at the array inlet to further into the array. The undulations start with higher amplitudes and lower frequency whereas further on, the undulations

decrease in amplitude and the frequency is much higher. The frame rate was 9.83 fps and the exposure time was 100 ms. The video corresponds to the data shown in Figure 4B in the main text.

Video S4. High-magnification (100x) fluorescent videographs of the migration of 166 kbp long DNA strands at 21 μm/s (3.5 mbar) in device #1. These videographs correspond to Figure 6A-C in the main text. The running buffer was 5x TE and 3% BME. The frame rate was 47.5 fps and the exposure time was 19.3 ms. The video corresponds to the data shown in Figure 6 in the main text.

9. References

1. Iarko, V.; Werner, E.; Nyberg, L.K.; Müller, V.; Fritzsche, J.; Ambjörnsson, T.; Beech, J.P.; Tegenfeldt, J.O.; Mehlig, K.; Westerlund, F.; et al. Extension of nanoconfined DNA: Quantitative comparison between experiment and theory. *Physical Review E - Statistical, Nonlinear, and Soft Matter Physics* **2015**, *92*, 1-8, doi:10.1103/PhysRevE.92.062701.
2. Rubinstein, M.; Colby, R.H. *Polymer Physics*; Oxford University Press: New York, 2003.
3. Reisner, W.; Pedersen, J.N.; Austin, R.H. DNA confinement in nanochannels: Physics and biological applications. *Reports on Progress in Physics* **2012**, *75*, doi:10.1088/0034-4885/75/10/106601.
4. Doi, M.; Edwards, S.F. *The Theory of Polymer Dynamics*; Oxford University Press, Inc.: New York, 1986; Volume 73.
5. Huang, L.R.; Cox, E.C.; Austin, R.H.; Sturm, J.C. Continuous Particle Separation Through Deterministic Lateral Displacement. *Science* **2004**, *304*, 987-990, doi:10.1126/science.1094567.
6. Chen, Y.; Abrams, E.S.; Boles, T.C.; Pedersen, J.N.; Flyvbjerg, H.; Austin, R.H.; Sturm, J.C. Concentrating genomic length DNA in a microfabricated array. *Physical Review Letters* **2015**, *114*, 1-5, doi:10.1103/PhysRevLett.114.198303.
7. Wunsch, B.H.; Kim, S.-C.; Gifford, S.M.; Astier, Y.; Wang, C.; Bruce, R.L.; Patel, J.V.; Duch, E.A.; Dawes, S.; Stolovitzky, G. Gel-on-a-chip: continuous, velocity-dependent DNA separation using nanoscale lateral displacement. *Lab on a Chip* **2019**, *19*, 1567-1578.
8. Huang, L.R.; Tegenfeldt, J.O.; Kraeft, J.J.; Sturm, J.C.; Austin, R.H.; Cox, E.C. A DNA prism for high-speed continuous fractionation of large DNA molecules. *Nature Biotechnology* **2002**, *20*, 1048-1051, doi:10.1038/nbt733.
9. Doggett, N.A.; Smith, C.L.; Cantor, C.R. The effect of DNA concentration on mobility in pulsed field gel electrophoresis. *Nucleic acids research* **1992**, *20*, 859-864.
